# Quantitative age grading of mosquitoes using surface‐enhanced Raman spectroscopy

**DOI:** 10.1002/ansa.202100052

**Published:** 2021-11-19

**Authors:** Danhui Wang, Jason Yang, Janam Pandya, John M. Clark, Laura C. Harrington, Courtney C. Murdock, Lili He

**Affiliations:** ^1^ Department of Food Science University of Massachusetts Amherst Massachusetts USA; ^2^ Department of Veterinary and Animal Sciences University of Massachusetts Amherst Massachusetts USA; ^3^ Department of Entomology College of Agriculture and Life Sciences Cornell University Ithaca New York USA

**Keywords:** age, mosquitoes, SERS, quantitative

## Abstract

Mosquito‐borne pathogens, including malaria, Zika, dengue, and chikungunya continue to be a major public health concern globally. Based on the understanding that only older female mosquitoes are infectious and represent a risk to human health, scientists have sought to age‐grade mosquitoes for decades. To date, however, no reliable, cost‐effective and practical methods exist to age older mosquitoes despite the tremendous epidemiological value of this approach. This study is the first attempt to develop a surface‐enhanced Raman spectroscopic (SERS) method to age mosquitoes. The water extracts of *Aedes aegypti* mosquitoes aged 0–22 days were mixed with silver nanoparticles. The SERS spectra, which were analysed by principal component analysis and partial least square (PLS), demonstrated the capability of this approach to predict the calendar age of mosquitoes between 0 and 22 days with the coefficient of correlation (*R*) = 0.994 and 0.990 for PLS model calibration and validation, respectively. Spectral analysis with both SERS and infrared spectroscopy revealed the key biological sources leading to changes in spectra allowing mosquito age‐grading is adenine‐containing compounds and proteins. In addition, we evaluated the impact of two arthropod‐borne pathogen deactivating pre‐treatments (bleach and ethanol) on the discrimination capability of the SERS approach. The result shows the ethanol treatment has the potential to enhance the discrimination capability and the safety of the approach. This study represents the first step towards developing the SERS approach as a quick, reliable and field‐deployable method for mosquito age‐grading, which would significantly improve the effectiveness of vector‐borne disease monitoring and prevention.

## 1 INTRODUCTION

A global resurgence of pathogens transmitted by mosquitoes is of great concern to public health. Yellow fever has re‐emerged in Africa in 2016, and dengue, which impacts the lives of an estimated 390 million people annually, takes the lives of thousands (primarily children) each year.^1^ Additionally, new threats that cycled silently in their sylvatic backdrops, such as chikungunya and Zika viruses, have surged forward and crossed hemispheres with a vengeance. This resurgence has resulted in over half of the world's population being at risk for mosquito‐borne diseases. Finally, despite decades of intervention efforts, malaria infections still cause about half a million deaths per year.^1^ The most substantial gains in the fight against these diseases have primarily been through vector control. These efforts, however, are threatened by the evolution of resistance (both chemical and behavioural) to commonly used insecticides and prohibitive costs and labour. Thus, considerable effort has been made to explore novel ways of controlling mosquito vectors.

As new vector control strategies are developed, there is a critical need to also develop more rapid methods for detecting the impact of control strategies on both mosquito vector survival and mosquito infection rates. The latter will be especially critical as testing of new control methods is reframed in the context of evidence‐based testing, with the rigour of clinical trials.^2^ Nonetheless, we find ourselves lacking the tools for making critical point‐of‐control decisions. For example, only older mosquitoes that have survived the extrinsic incubation period of a vector‐borne pathogen are competent to transmit to humans when they ingest a blood meal. A large portion of the mosquito population is, therefore, unable to transmit many vector‐borne pathogens, with very old mosquitoes being disproportionately important for transmission. Despite this well‐known concept, we lack tools to age mosquitoes in the field, especially at the critical point where they become a risk to humans (e.g., malaria transmission risk is associated with vector age >10 days).^3^ Methods such as detecting follicular relics have been around for decades as a tool for determining rough physiological age (number of egg clutches produced by females), but these are labour‐intensive and do not work well in *Aedes* mosquitoes.^4^ Prior efforts have utilised cuticular hydrocarbon profile,^5^, ^6^ or gene expression changes^7^, ^8^ to attempt mosquito ageing to this point without success. In addition, these latter two methods are expensive and require specialised equipment and expertise, making them impractical for realistic use.

Spectroscopic techniques have gained great attention due to their rapid data acquisition capabilities with minimum sample pre‐treatment. The applications of infrared (IR) spectroscopy, including near IR (NIR) and mid‐IR (MIR), for differentiating a variety of mosquito traits (age, species and infection status) have been reported.^4^ NIR measures the absorption of organic compounds within a sample and reports an electromagnetic spectrum in the NIR region (350–2500 nm) and MIR measures the range between 2500 and 25,000 nm. With chemometrics, NIR techniques are able to classify mosquitoes into binary age categories (e.g., less than and greater than 7 days old).^9^, ^10^, ^11^, ^12^, ^13^, ^14^, ^31^ However, this binary classification cannot directly indicate malaria transmission risk, which is associated with vector age >10 days.^3^ Krajacich et al. evaluated the regression and classification models using NIR on datasets of wild *Anopheles gambiae* reared from larvae collected from the field. The accuracy for determining the wild‐caught larvae reared was low (69.6%).^16^ The MIR method can only differentiate between very young (1 day) and very old (15 days).^17^ This technique has not been validated in the field.^18^

Here we demonstrate that a surface‐enhanced Raman spectroscopic (SERS) method has the potential to solve many of the most significant obstacles in age‐grading mosquitoes. This method is the first attempt to use the SERS technique to monitor the changes in the biomolecules extracted from mosquitoes to predict the calendar age of mosquitoes in a non‐binary classification manner, which includes older epidemiologically important mosquitoes. Raman spectroscopy measures characteristic molecular vibrations non‐destructively and rapidly. Compared with IR spectroscopy, the Raman bands are more distinct and carry richer information in general.^19^ With certain metallic nanosubstrates, such as silver nanoparticles (AgNPs), Raman scattering is greatly enhanced, making this SERS technique ultrasensitive. In addition, the unique selectivity based on the interactions between the analyte and NPs increases the robustness of SERS as an analytical technique for complex biomaterial analysis.^18^, ^19^ Laboratory‐reared adult female Thai strain *Ae. aegypti* (age: 0–22 years) were used as a model mosquito in this study for the demonstration of the age‐grading capability of SERS. Different inactivation pre‐treatments commonly used to kill bacteria and viruses were evaluated for their impact for SERS analysis. Principal component analysis (PCA) and partial least square (PLS) were employed for spectral characterisation and age prediction. In addition, IR spectroscopy was applied to provide more information regarding the biomolecule sources leading to changes in SERS spectra that allow mosquito age‐grading. If successful, this approach would be a game‐changer for vector‐borne disease monitoring and prevention.

## 2 MATERIALS AND METHODS

### 2.1 Mosquito rearing

A Thai strain of Ae. aegypti originating from field‐collected populations in Bangkok, Thailand (15°72′N, 101°75′E) was used for all laboratory experiments. This colony has been supplemented annually with F1 eggs since 2009 to maintain natural levels of genetic heterogeneity. In brief, mosquitoes were reared and held in an environmental chamber at 28 ± 0.27°C and 85.09 ± 4.60% relative humidity (RH) as previously described to obtain medium body‐sized adults.^22^ After eclosion, adults were held in 8 L bucket cages with 10% sugar water to obtain known calendar ages of 0, 3, 6, 10, 12, 14, 18 and 22 days using previously described methods.^23^ ^–^ ^24^ Three batches of independently‐reared mosquitoes from each age cohort were frozen and shipped on ice from Cornell to the University of Massachusetts.

### 2.2 Chemicals and materials

AgNPs were synthesized using a previously published method based on citrate reduction.^23^, ^24^ Chemicals including hydroperoxide (H_2_O_2_), ethanol (CH_3_CH_2_OH) and bleach (Sodium hypochlorite, 10%) were purchased from Fisher Scientific (Waltham, MA).

### 2.3 Mosquito sample preparation for UV‐Visible, SERS, and Fourier Transform IR analysis

Mosquitoes were thawed immediately before use. For sample preparation, one mosquito was immersed in 200 μl Milli‐Q water and homogenised using an ultrasonic disruptor (Branson; Fisher Scientific) for 3 min. The homogenates were centrifuged at 9000 x g for 5 min at 4°C to separate the water‐soluble components (middle layer) from the fat (top layer) and tissue debris (bottom layer). The middle layer (100 μl) was carefully collected, diluted with 400 μl Milli‐Q water and mixed with 500 μl of the synthesized AgNP colloidal solution for 15 min.

For UV‐Visible (UV‐Vis) analysis, 1 ml aliquot of either amended AgNPs (AgNPs mixed with mosquito water extracts) or un‐amended AgNPs was analysed using SpectraMax Microplate Readers (Molecular Devices, San Jose, CA), respectively.

For SERS analysis, a 5 μl aliquot of amended and un‐amened AgNPs was deposited onto an aluminium foil‐covered glass slide and dried for SERS analysis.

For attenuated total reflection‐Fourier transform IR (ATR‐FTIR) analysis, amended AgNPs were recovered using centrifugation (9000 x g for 5 min at 4°C) and rinsed with 1 ml Milli‐Q water to remove water‐soluble bounded compounds. Note that, 1 ml aliquot of a 10% H_2_O_2_ solution was then added to water‐rinsed AgNPs and incubated for 15 min at room temperature. Following incubation, visually all AgNPs were digested and 20 μl aliquot was deposited onto the surface of a horizontal ATR crystal for spectroscopic analysis.

### 2.4 Inactivation pre‐treatments

To evaluate the impact of inactivation pre‐treatment used commonly to kill bacteria or viruses, 3, 10 and 18 days old mosquitoes were immersed in 70% ethanol or 10% bleach for 10 min, respectively, and then they were rinsed with Milli‐Q water. After that, the same protocols described above were applied to obtain amended AgNPs for SERS analyses.

### 2.5 Raman instrumentation and data analysis

SERS spectra were obtained using a DXRi Raman microscope (Thermo Fisher Scientific, Madison, WI) with a 780 nm laser and a 20x objective. The laser power was set to be 5 mW and the acquisition time was 2 s. For spectral variation analysis, 30 spectra were collected from the newly eclosed adult (day 0) mosquito sample. To analyse each age group, three mosquitoes were randomly chosen, and 6–8 spectra were collected from each mosquito. The experiment was repeated three times using three independent batches of mosquitoes and AgNPs.

Data analyses were performed using PCA and PLS regression models, where a data set from one batch was imported to TQ analyst software (Thermo Fisher Scientific). Data pre‐processing, such as standard normal variate and second derivative transformation with Norris derivative filter (segment length, 7 and gap between segments, 7), were applied. For PCA, 10 PCs were used for calculation and 3D‐PCA plots were drawn to visualise the data separation in each age group. PLS model calibration used 75% randomly selected data for model calibration and the remaining 25% for validation. Correlation coefficient (*R*) and root mean square error of calibration (RMSEC) and root mean square error of prediction (RMSEP) were calculated for evaluating the quality of the models.

### 2.6 ATR‐FTIR instrumentation and data analysis

IR spectra were recorded on a horizontal diamond ATR element with an FTIR Spectrophotometer (IRTracer‐100; Shimadzu, Kyoto, Japan). The scans were performed in the wavelength range of 145–3500/cm with a resolution of 16/cm. Twenty scans were accumulated per spectrum. IR spectra were analysed using PeakFit software (Systat Software Inc, San Jose, CA, USA) in which the deconvolution of amide peaks to individual peaks was performed using a Gaussian response function with a Fourier deconvolution algorithm.

## 3 RESULTS

### 3.1 Characterisation of SERS spectra obtained from *Ae. aegypti* water‐based extract

**FIGURE 1:**
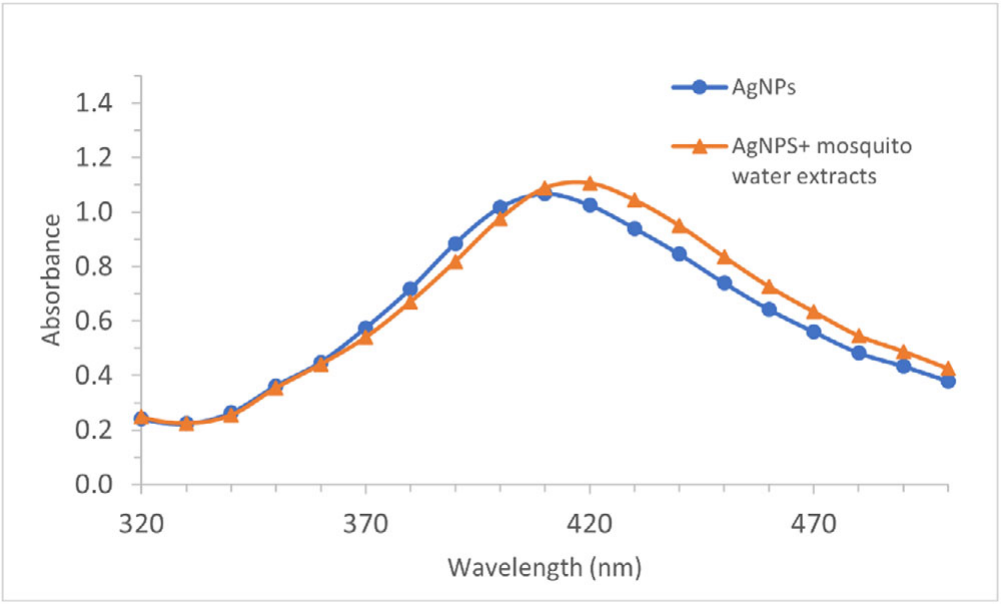
UV‐Visible (UV‐Vis) spectra of silver nanoparticles (AgNPs) (blue dots) and the mixture of AgNPs and mosquito water extracts (orange triangles)

**FIGURE 2:**
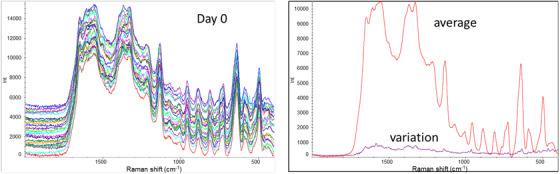
Surface‐enhanced Raman spectroscopic (SERS) spectra (*N* = 30) collected from the dried spot of silver nanoparticles mixed with the water extraction of mosquitoes reared at day 0. The average and variation spectra demonstrate the great consistency of the data

**FIGURE 3:**
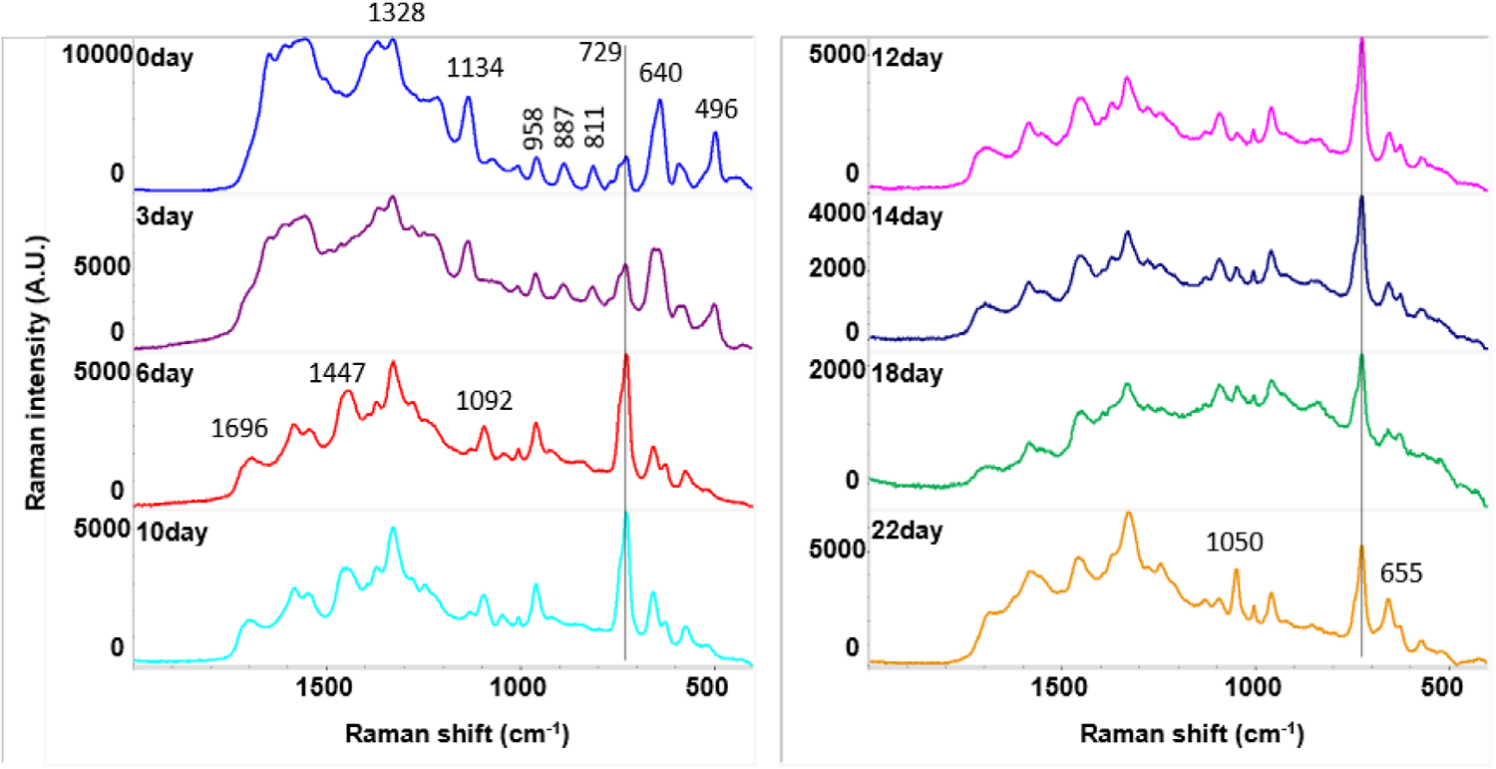
Surface‐enhanced Raman spectroscopic (SERS) spectra of *Ae. aegypti* extract from adults with calendar ages of 0, 3, 6, 10, 12, 14, 18 and 22 days

**FIGURE 4:**
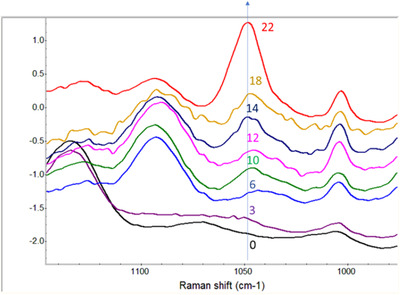
The 1050 peaks from the spectra of mosquitoes aged from days 0 to 22, demonstrating an age‐specific trend

After mixing the water extracts of mosquitoes with AgNPs (amended AgNPs), the UV‐Vis absorbance of un‐amended AgNPs shifted from 410 to 420 nm (Figure 1), which demonstrates an interaction between AgNPs and biological compounds in the water extracts. The recovered AgNPs colloids had a uniform drying pattern on the aluminium foil‐covered glass slide, and the resulting SERS spectra exhibited excellent consistency with a variation of less than 5% (Figure 2). With this method, we obtained SERS spectra from different age groups of *Ae. aegypti* to determine the feasibility of using SERS to age mosquitoes. As shown in Figure 3, SERS spectra of *Ae. aegypti‐*amended AgNPs contain a variety of distinct peaks in the range of 400–1800/cm. Among the many peaks that showed changes over the age groups, the peak at 729/cm is most noticeable and is characteristic of adenine‐containing compounds, a well‐known SERS biomarker.^27^ ^,^ ^28^ Visually, there are clear differences between younger groups (days 0 and 3), older ones (days 6–18) and the oldest group (day 22). The spectra of younger groups had relatively low intensities for the 729/cm peaks, but significantly higher intensities for peaks at 640 and 496/cm, which are assigned to the stretching mode of carbon‐sulfur (C‐S) and disulfide (S‐S) bonds, respectively.^29^ ^,^ ^30^ Additional features at 887 and 881/cm and in the range between 1500 and 1600/cm were also observed for younger groups. The older groups (days 6–18) had distinct features at 1696/cm (amide I), 1447/cm (CH_2_/CH_3_ deformation) and 1092/cm (C‐C skeletal and C‐O‐C stretching) and a clear trend of decreasing intensities for the 729 and 1092/cm peak. The 1050/cm peak, which is assigned to C‐N stretching, had a noticeable decreasing intensity trend from days 0 to 22 (Figure 4). In addition, the day 22 spectra had significantly higher intensity peaks at 1050 and 655/cm. For more details, the peak assignment table for spectra obtained at days 3, 10 and 18 (Figure S1) can be found in Table S1. The above experiment was repeated three times independently, based on different batches of AgNPs, different batches of mosquitoes and on different days, resulting in very consistent spectral patterns (Figure S2).

To further characterise the biochemical make‐up of molecules that bind on the AgNPs, we treated amended AgNPs with H_2_O_2_ and analysed these samples using ATR‐FTIR. The resulting IR spectra clearly show amide peaks, which is indicative of protein binding on the AgNPs (Figure S3). After deconvoluting the amide peaks, the composition of the proteins found in the 0th, 10th and 18th‐day samples was significantly different. This result indicates that age‐specific proteins are bound on the AgNPs and may likely provide the basis for age‐grading.

### 3.2 Age‐grading of *Ae. aegypti* using PCA and PLS regression models

**FIGURE 5:**
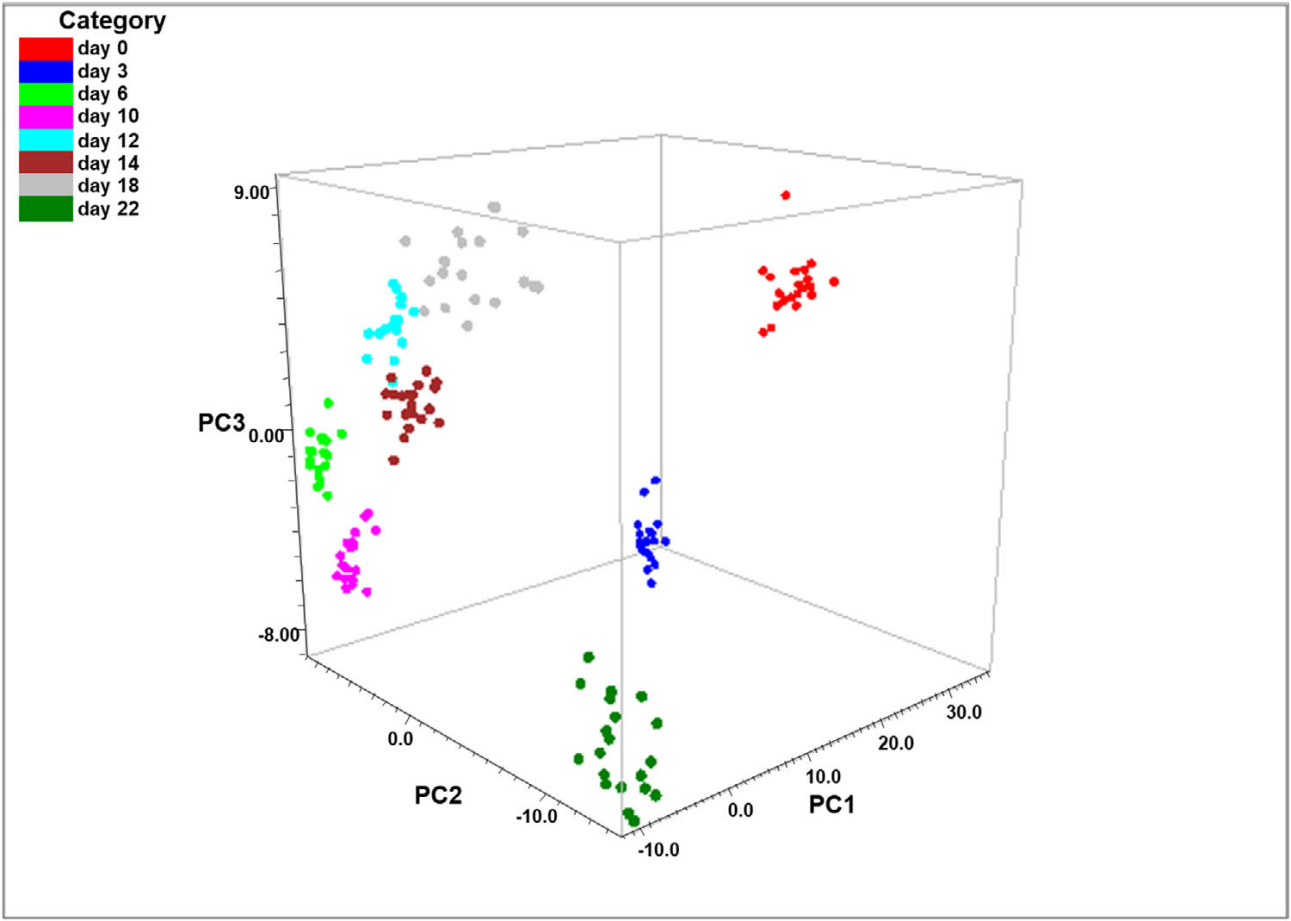
Principal component analysis (PCA) plot of spectra of the water extracts of *Ae. aegypti* on different days

PCA was applied to further analyse the spectral variances among different days. The 3D PCA plot of the entire data set appears in Figure 5. The overall PCA captured 89.45% of the variance with three PCs, and 96% of the data points were classified accurately. The 2D PC1–PC2 and PC2–PC3 plots can be found in Figure S4. The data points of each day were highly clustered and can be discriminated from each other. Along the PC1, days 0 and 3 were well separated from the rest. Along the PC2 and PC3, days 6–22 were clearly separated. The PCA plots demonstrated the capability of discrimination between different age groups, which is superior to the current binary discrimination by NIR and MIR techniques.

**FIGURE 6:**
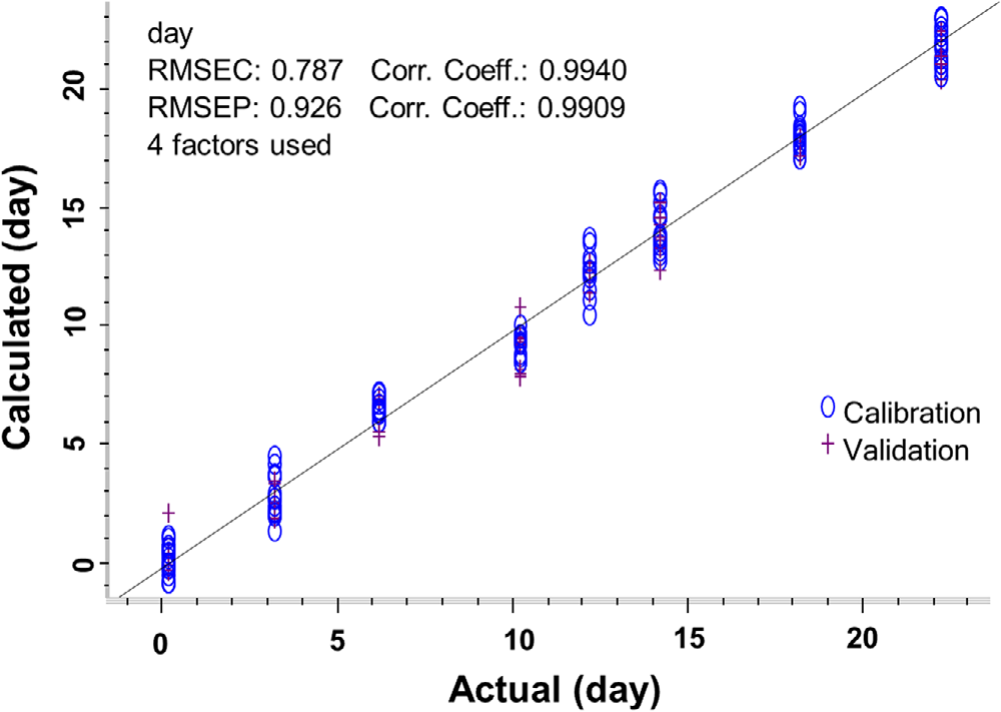
Partial least square (PLS) regression models of water extracts of *Ae. aegypti* on different days. (A) Calibration model, (B) Cross‐validation model using the leave‐one‐out method

To evaluate the capability of quantitative age prediction for mosquitoes between 0 and 22 days old, we analysed the SERS spectra using PLS regression (Figure 6). The differences between actual and predicted values appear in Figure S5. The PLS regression calibration model reached an *R* of 0.994, indicating a very strong positive relationship between the age determined by the model and the actual calendar age of mosquitoes from 0‐22 days. The RMSEC is 0.787. The validation model had a very high *R* of 0.990, and a low RMSEP of 0.926, which demonstrates the reliability of the prediction model.

### 3.3 Impacts of inactivation treatments on SERS analysis

**FIGURE 7:**
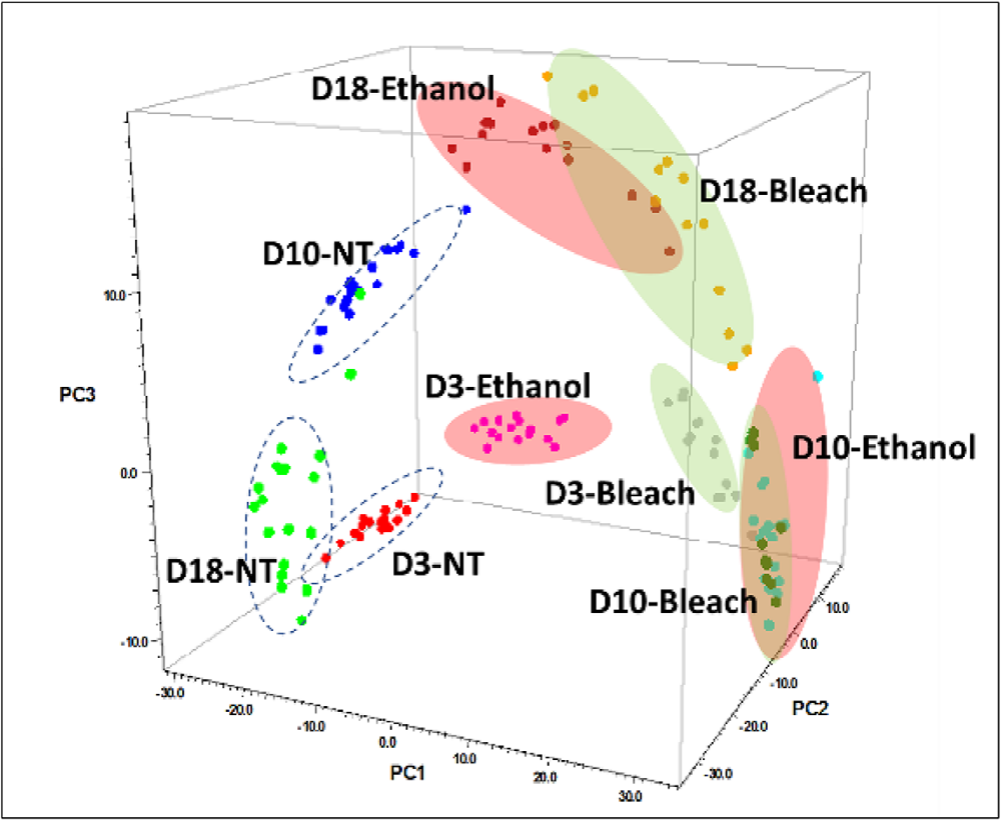
Principal component analysis (PCA) plot of spectra of the water extracts of *Ae. aegypti* at different days with and without treatment. D3, 10 and 18 refer to the age of mosquitoes. NT: not treated

For further developing this approach to be a field‐deployable method, one important consideration is the safety of sample handling. Here we evaluated the impact of two pathogen deactivating agents, 70% ethanol and 10% bleach, on SERS analysis of mosquitoes using mosquitoes of days 3, 10 and 18 as models. The results show after ethanol or bleach treatment, the major SERS peaks were still observed with some minor degrees of a shift in wavelength and/or intensity, and a few additional new peaks were found in treated spectra (Figure S6). More importantly, as demonstrated in the PCA plot (Figure 7), the capability to differentiate between different ages was still maintained after treatment, with the ethanol treatment exhibiting better data separation than the bleach treatment and no treatment (control). This result indicates that the involvement of an inactivation step may facilitate safe sample handling, age‐grading and ultimate field testing in future applications. More research on the validation of the viral inactivation and optimisation of the PLS models will be conducted in the future.

## 4 CONCLUSION

In conclusion, we have demonstrated the following: 1) The SERS approach differentiates *Ae. aegypti* of different ages based on the fact that age‐specific biological compounds (e.g. adenine‐containing compounds, proteins) bind onto AgNPs, 2) SERS in combination with PCA and PLS allows the development of prediction models where the predicted calendar age of *Ae. aegypti* is highly correlated to the actual age and 3) The inactivation treatment protocol has the potential to enhance the capability of SERS to age‐grade *Ae. aegypti* as well as allow the safe handling of field‐collected samples. This study represents the first step in developing SERS as a novel technology for determining mosquito's age in the field with the potential to dramatically improve the ability to investigate transmission dynamics and age‐related risk of mosquito‐borne pathogens in nature. Future experiments will be needed to investigate the impact of environmental variation in key abiotic (e.g., temperature) and biotic (e.g., diet) traits that can impact the physiological age of mosquitoes^32^ on the SERS models. In addition, the capability of SERS models for determining the infection status of *Ae. aegypti* will be investigated. The long‐term goal is to establish a rapid, effective, and field‐deployable method and user‐friendly web or APP‐based prediction models to detect and analyse the status and public health risk of field‐collected mosquitoes rapidly to facilitate point‐of‐control interventions.

## CONFLICT OF INTEREST

The authors declare that they have no conflict of interest.

## Supporting information

Supporting Information

## Data Availability

The data that support the findings of this study are available from the corresponding author upon reasonable request.
