# Supplementary material for: Quantitative age grading of mosquitoes using surface‐enhanced Raman spectroscopy
Source: Anal Sci Adv. 2021 Nov 19;3(1-2):47–53. doi: 10.1002/ansa.202100052 (PMC10989641; doi:10.1002/ansa.202100052)
Supplement: Supplementary file 1 — Supporting Information [file ANSA-3-47-s001.docx]

**Supporting information**

**Age grading of mosquitoes using surface-enhanced Raman spectroscopy**

Danhui Wang^1^, Jason Yang^1^, Janam Pandya^1^, John Clark^2^, Laura Harrington^3^, Courtney Murdock^3^, Lili He^1*^

1 Department of Food Science, University of Massachusetts, Amherst, Massachusetts 01003, United States

2 Department of Veterinary and Animal Sciences, University of Massachusetts, Amherst, Massachusetts 01003, United States

3 Department of Entomology, College of Agriculture and Life Sciences, Cornell University, Ithaca, NY, United States

*Corresponding Author

Telephone: +1-413-545-5847. E-mail: [lilihe@umass.edu](mailto:lilihe@umass.edu).


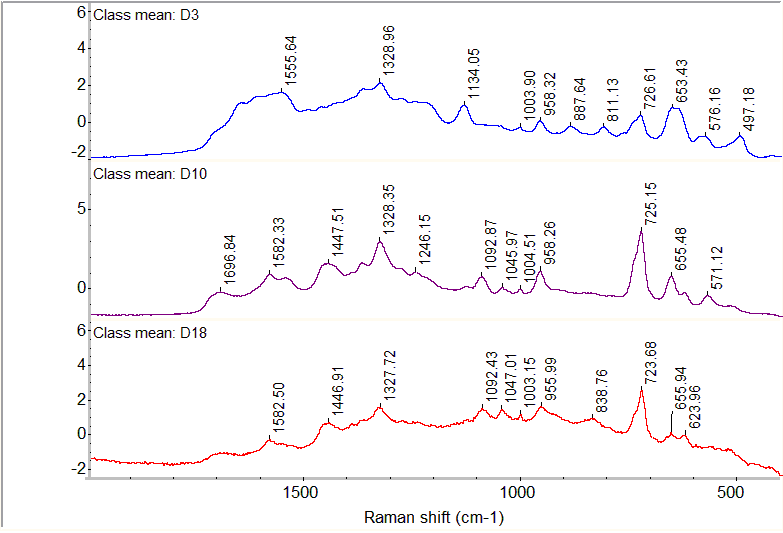


Figure S1. SERS spectra of mosquitoes at day 3, 10, and 18 and their peak shifts.

Table S1. Peak assignment of the SERS spectra of mosquitoes at day 3, 10, and 18.

|  | D3 | D10 | D18 | Peak assignment |
| --- | --- | --- | --- | --- |
| 1696 | x | x | x | Amide I |
| 1582 | x | x | x | phenylalanine, hydroxyproline, tyrosine |
| 1447 |  | x | x | CH2/CH3 deformation |
| 1328 | x | x | x | CH2/CH3 wagging |
| 1134 | x |  |  | C-C/C-N stretching |
| 1093 |  | x | x | C-C skeletal and C-O-C stretching from glycosidic link |
| 1046 |  | x | x | C-N stretching |
| 1003 | x | x | x | phenylalanine |
| 958 | x | x | x | phosphate in nucleic acid, C=C deformation |
| 887 | x |  |  | hydroxyproline |
| 839 |  |  | x | polysaccharides |
| 811 | x |  |  | Tryptophan |
| 726 | x | x | x | hypoxanthine or adenine in FAD and NAD |
| 655 | x | x | x | C-S stretching |
| 623 |  | x | x | C-C twisting |
| 576 | x |  |  | tryptophan |
| 571 |  | x |  | C-S-S-C |
| 497 | x |  |  | S-S disulfide stretching |


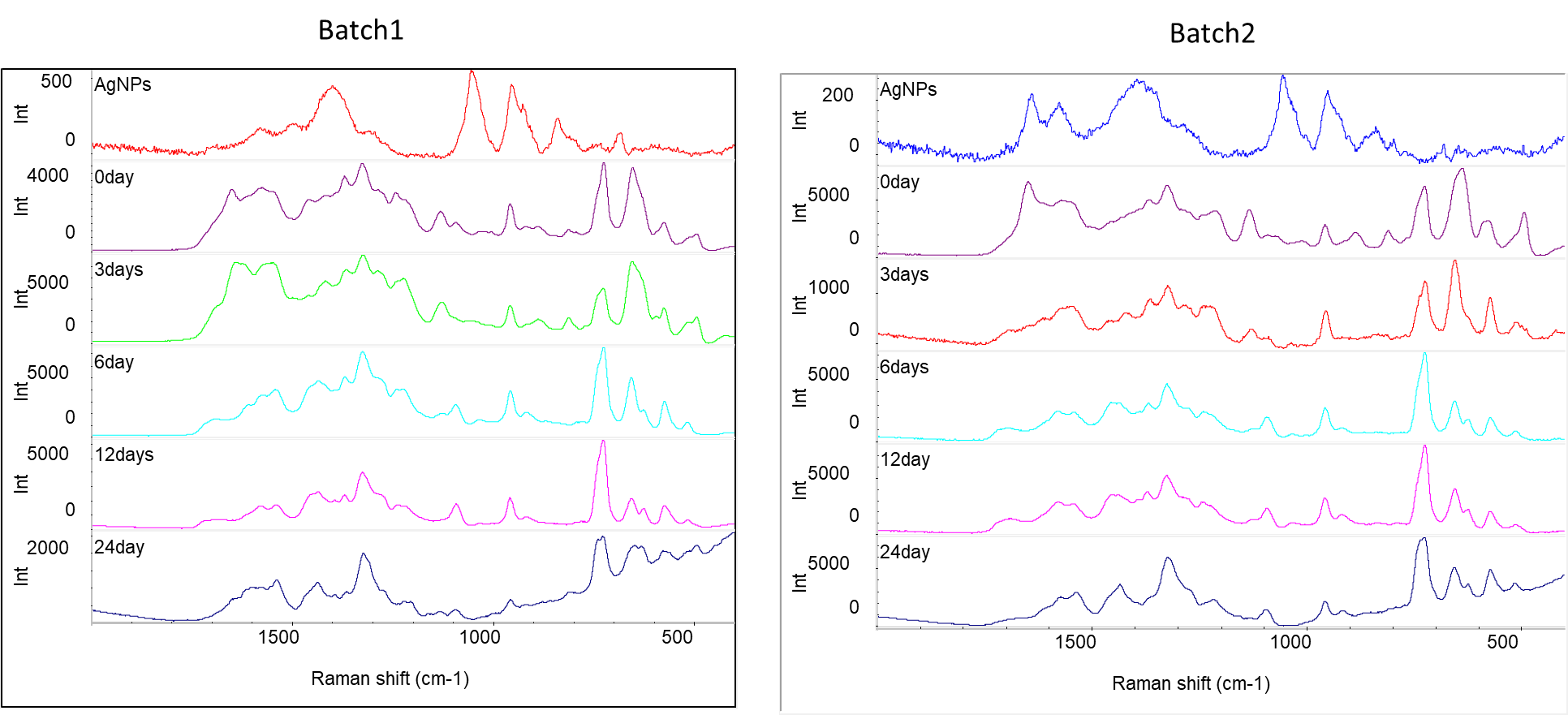


Figure S2. The other 2 sets of SRES spectra collected from independently reared mosquitoes at day 0- 24 using silver nanoparticles synthesized at different days. The results demonstrated the reproducibility of this approach.


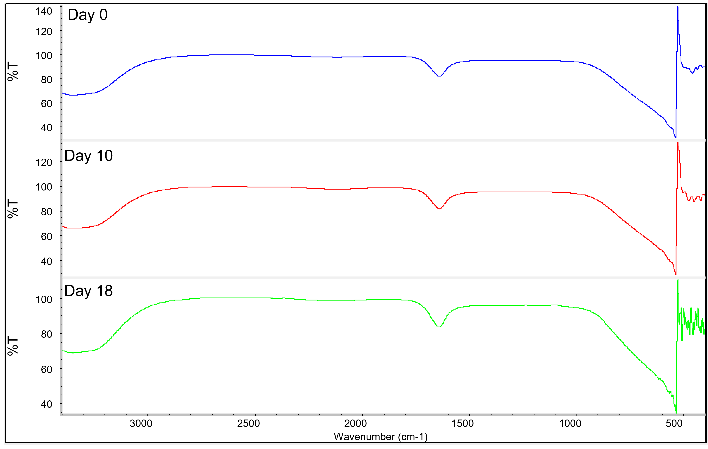

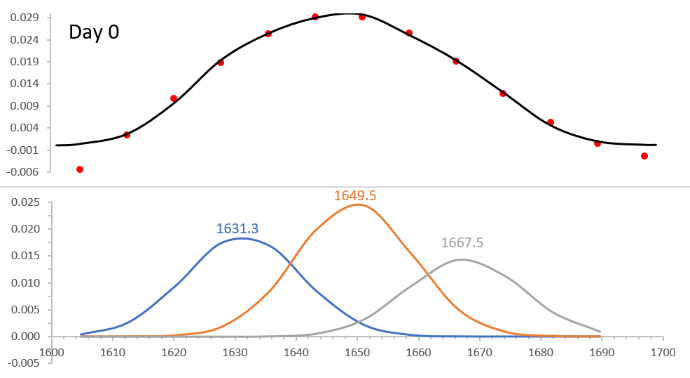


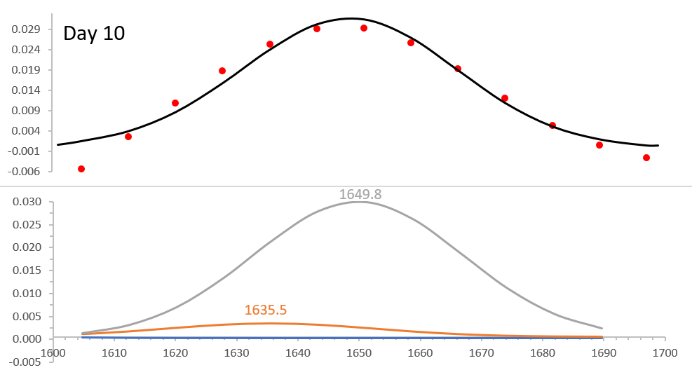

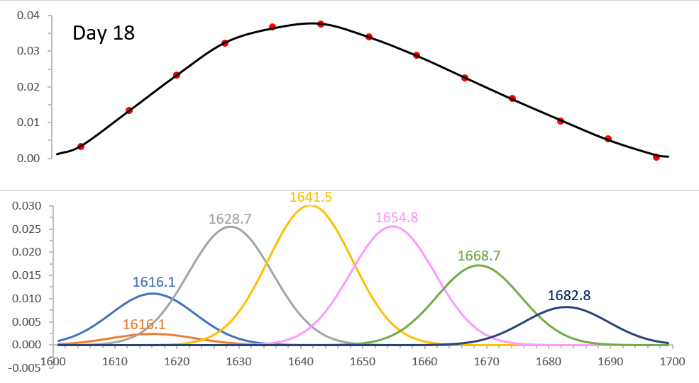


Figure S3. IR spectra of digesta of AgNPs and mosquito water extracts with H_2_O_2_ at day 0, day 10, and day 18 respectively. Deconvolution of the IR spectra of the digesta of day 0, day 10, and day 18.


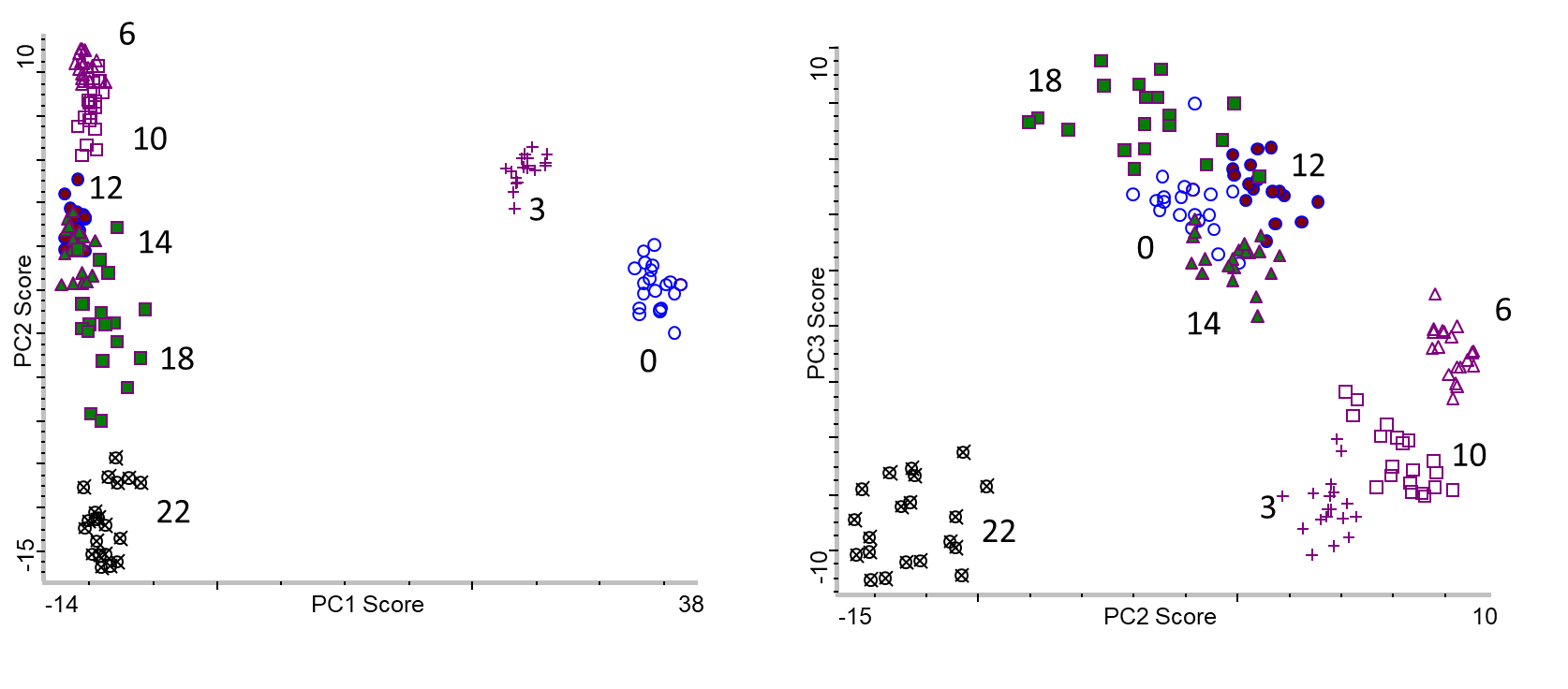


Figure S4 2-dimennsional PCA plots of the entire data set, PC1 and PC2, PC2 and PC3.


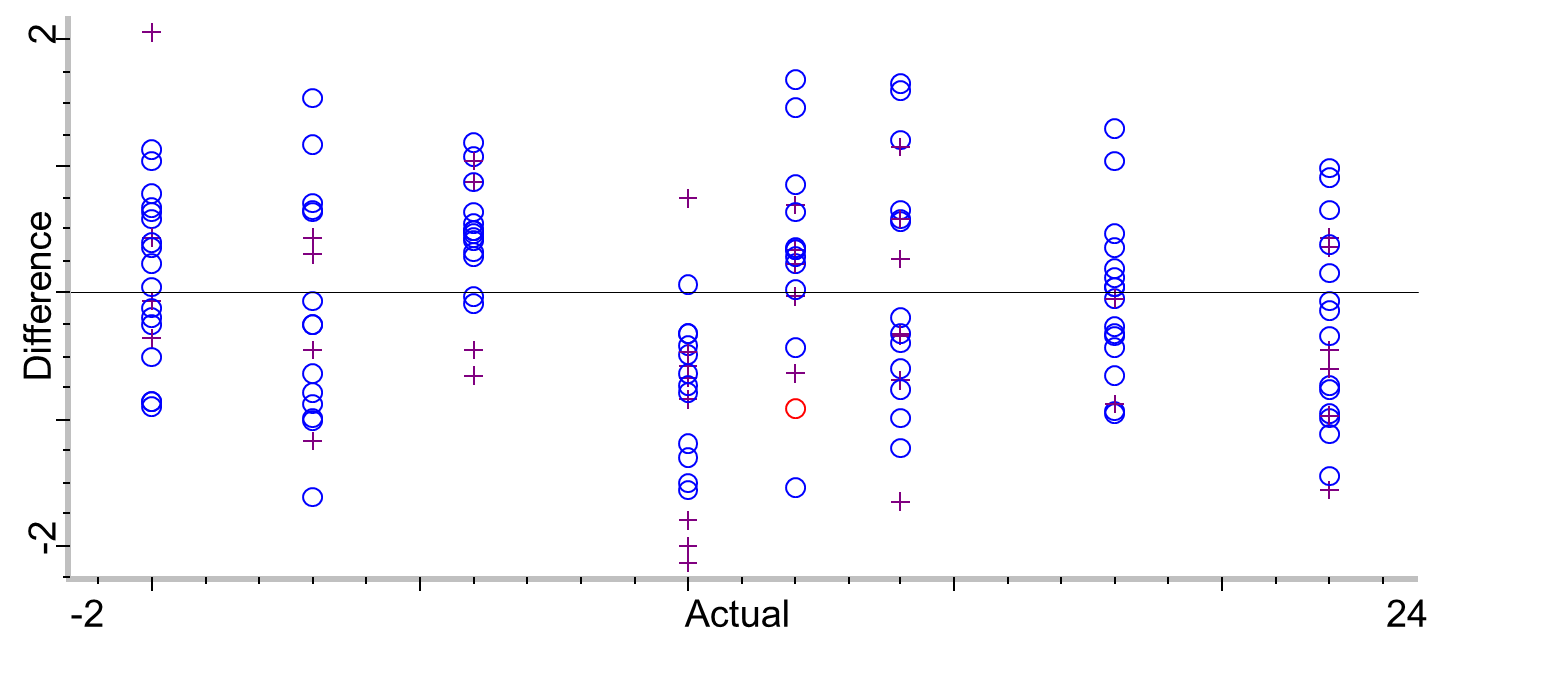


Figure S5 The differences between actual and predicted values of the PLS models.


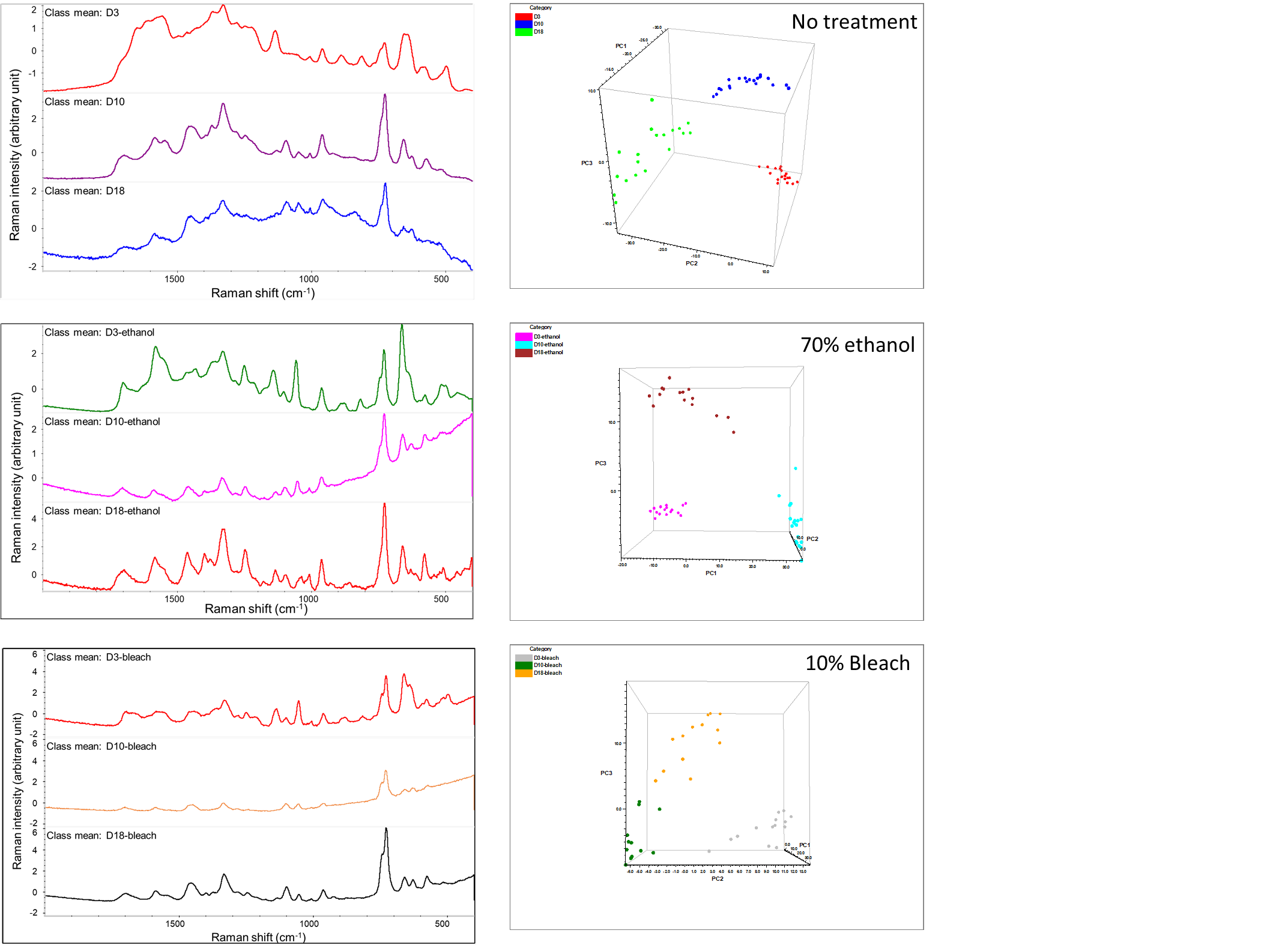


Figure S6. SERS spectra and PCA plots of the SERS spectra from untreated, ethanol-treated, and bleach-treated groups (day 3, 10, and 18).
